# Hallucinogen use is associated with mental health and addictive problems and impulsivity in university students

**DOI:** 10.1016/j.abrep.2019.100228

**Published:** 2019-10-18

**Authors:** Jon E. Grant, Katherine Lust, Samuel R. Chamberlain

**Affiliations:** a Department of Psychiatry & Behavioral Neuroscience, University of Chicago, Chicago, IL, USA; b Boynton Health Service, University of Minnesota, USA; c Department of Psychiatry, University of Cambridge, UK; d Cambridge and Peterborough NHS Foundation Trust (CPFT), UK

**Keywords:** Hallucinogens, Illicit, Drugs, Addiction, Impulsivity

## Abstract

**Background:**

This study examined the prevalence of hallucinogen use in a large sample of university students and its associations with mental health issues.

**Methods:**

9449 students received a 156-item anonymous online survey, which assessed the use of hallucinogens (ever or past year), alcohol and drug use, mental health issues, and impulsive and compulsive traits. Group differences were characterized using statistical tests (p values reported uncorrected, but only regarded as significant if surviving Bonferroni correction).

**Results:**

3525 university students (57.7% female) responded to the survey. The prevalence of past 12-month hallucinogen use in the sample was 4.7%, with an additional 6.4% reporting having used more than 12 months ago. Hallucinogen use was associated with the use of multiple other drugs (e.g., alcohol, opiates) (each p < 0.001), mental health problems (p < 0.001), risky sexual behavior (p < 0.001), low self-esteem (p = 0.004), and impulsivity traits (p < 0.001) but not compulsivity. Effect sizes were small to medium.

**Conclusion:**

Past use of hallucinogens was reported in 11.1%, and was associated with a variety of mental health and drug use problems. Clinicians should be aware that use of hallucinogens is common and mental health problems are more likely in those who use hallucinogens. This study indicates the need for longitudinal research into the negative effects of hallucinogen use on brain function and mental health, especially in young people. Such research should address the extent to which impulsive traits predispose to various substance use problems, versus the direct effects of hallucinogens (and other substances) on mental health.

## Highlights

• Among university students, lifetime use of hallucinogens was reported as 11.1%.
• Young adults who use hallucinogens have problems with a range of addictive substances and unhealthy behaviors.
• Elevated impulsivity is a common cognitive/personality feature underlying the problems associated with hallucinogen use.

## 1 Introduction

Plant-based hallucinogens have been used throughout the world for thousands of years (Bruhn, de Smet, El-Seedi, & Beck, 2002). In recent years, there is a renewed interest in several hallucinogens as novel agents to treat psychiatric disorders – such as psilocybin for treatment of substance use disorder or refractory depression (Bogenschutz et al., 2015, Carhart-Harris et al., 2017); or MDMA for post-traumatic stress disorder or social anxiety disorder (Danforth et al., 2016, Sessa, 2017). While apparent positive benefits of such substances on aspects of mental health have been reported by some researchers (Hendricks, Thorne, Clark, Coombs, & Johnson, 2015), there is a long history of adverse reactions to many of these substances reported in the psychiatric literature, e.g. (Horowitz, 1969, Ungerleider et al., 1968).

The term “Hallucinogen” in the Diagnostic and Statistical Manual Version 5 (DSM-5) refers to a large category of psychedelic drugs that produce similar alterations of perception, mood and cognition (American Psychiatric Association, 2013). These substances include psilocybin, mescaline and lysergic acid diethylamide (LSD), the NMDA antagonist phencyclidine (PCP), 3,4-Methylenedioxy-methamphetamine (MDMA), and Salvia divinorum (American Psychiatric Association, 2013). Recent data from the National Epidemiologic Survey on Alcohol and Related Conditions (NESARC) (n = 36,255) found that 12-month and lifetime prevalence rates for hallucinogen use were 0.62% and 9.32%, respectively, with a mean age of onset of hallucinogen use of 17 years (Shalit, Rehm, & Lev-Ran, 2019). Given the long history of hallucinogen use throughout the world, and the data showing that use is fairly common today, questions remain as to whether and to what extent these substances are problematic for many people (Carbonaro et al., 2016).

Use of hallucinogens frequently presents alongside other substance use issues and mental health problems. Using the NESARC data, Shalit and colleagues reported that hallucinogen use was significantly associated with mood disorders, anxiety disorders (particularly PTSD), eating disorders, personality disorders, substance use disorders (particularly opiate use disorder), and past suicide attempts (Shalit et al., 2019). These data however are inconsistent with other studies that have failed to find mental health associations with hallucinogen use or in fact have found hallucinogen use to be potentially associated with lower mental health problems (Hendricks et al., 2015, Krebs and Johansen, 2013).

In view of the recent renewed interest in these substances and the inconsistent findings of mental health associations with hallucinogens, the current study sought to examine both the prevalence of the use of hallucinogens among university students; and to examine related behaviors and mental health issues. We included questionnaire-based measures of impulsivity and compulsivity, since these concepts have been implicated in different stages of addiction (Yucel et al., 2018). Based on the previous literature, we hypothesized that the use of hallucinogens would be associated with elevated rates of other substance use, mental health issues, trait impulsivity and compulsivity, riskier sexual practices, and academic impairments compared to students who do not use hallucinogens.

## 2 Material and methods

### 2.1 Survey design

Researchers at the Department of Psychiatry and Behavioral Neuroscience at the University of Chicago and Boynton Health Services at the University of Minnesota jointly developed the *Health and Addictive Behaviors Survey,* an online survey examining the use of alcohol, drugs, and mental health issues, in university students. All study procedures were conducted in accordance with the Declaration of Helsinki and the University of Minnesota’s Institutional Review Board approved the study.

### 2.2 Participants

10,000 undergraduate and graduate/professional students at a large Midwestern university were chosen randomly using a computer-generated selection with email addresses and sent an online survey during a three-week period in the Autumn of 2016. Of the 10,000 email invitations, 9449 were successfully received by the recipients. Of the 9449 students who received the invitation to participate, 3525 (37.3%) completed the survey, a response rate in keeping with other health surveys (Baruch and Holtom, 2008, van Horn et al., 2009).

The survey first presented students with information sheets about the study (including informing them that all information was anonymous and confidential). Students then provided consent to take part or opted out. Subsequent questions were only presented when informed consent had been provided. Students were informed that after completing the survey email address would be entered in to a raffle wherein 10 students would be randomly chosen to receive prizes: 3 would win tablet computers, 4 would win $250 gift certificates to an online retailer, 2 would win $500 gift certificates, and there would be a single winner of a $1000 gift certificate. To maintain anonymity, the email addresses were not linked to questionnaire responses. Participants were required to review all survey questions to be eligible for the prize drawings, but they were not required to answer all questions given the sensitive nature of some items.

### 2.3 Assessments

The survey consisted of 156 questions and took approximately 30 min to complete. Hallucinogen use was assessed by asking participants if they had used hallucinogens (e.g., LSD, MDA, MDMA [Ecstasy], Mushrooms, Peyote) in the past year or used ever in their lifetime. Participants were grouped into “current” hallucinogen use if they reported using any in the last 12 months, those who used hallucinogens previously, but not in last 12 months, were labeled as “past” hallucinogen use. Those who had never used hallucinogens comprised the third category.

The following demographic measures were collected: gender, year in college, and Grade Point Average (GPA). In addition to asking demographic, clinical, and sexual health information, the survey used measures of interest focusing on three domains: Drug and Alcohol Use; Mental Health Problems; and Impulsivity/Compulsivity:

#### 2.3.1 Drug and alcohol use

Participants were asked if they had ever used an illicit drug (binary); and were asked about whether they had used the following in the past 12 months (each a binary response): amphetamines, cocaine, heroin, hallucinogens, marijuana or hashish, prescription opioid pain medication, or sedatives. In addition to use of drugs and alcohol, participants were screened for possible problematic use by using the *Alcohol Use Disorders Identification Test (AUDIT)* (score of ≥8 indicating potentially harmful alcohol use (Saunders, Aasland, Babor, de la Fuente, & Grant, 1993); and the *Drug Abuse Screening Test (DAST-10)* (score of 3 indicating a positive screen for a drug use disorder) (Skinner, 1982, Yudko et al., 2007).

#### 2.3.2 Mental health problems

Participants were screened with the following reliable and valid measures; *Patient Health Questionnaire (PHQ-9)* (score of ≥10 indicating depressive symptoms of moderate or higher severity) (Kroenke, Spitzer, & Williams, 2001); *Generalized Anxiety Disorder 7 (GAD-7)* (score of 10 or greater indicating clinically significant anxiety) (Spitzer, Kroenke, Williams, & Lowe, 2006); *Primary Care PTSD Screen (PC-PTSD)* (score of ≥ 3 indicating probable posttraumatic stress disorder, PTSD) (Prins et al., 2003); *Adult ADHD Self-Report Scale (ASRS-v1.1) Part A* (6 questions screening for attention-deficit/hyperactivity disorder) (Kessler et al., 2005, Kessler et al., 2007); *Minnesota Impulsive Disorders Interview (MIDI)* (screens for binge eating disorder and gambling disorder) (Chamberlain and Grant, 2018b, Grant, 2008); and the *Rosenberg Self-Esteem Scale (RSES)* (score <15 indicating low self-esteem) (Rosenberg, 1965).

#### 2.3.3 Impulsivity/compulsivity

Impulsivity refers to a tendency towards inappropriate, premature, unduly hasty acts (Evenden, 1999); whereas compulsivity refers to a tendency towards repetitive habitual actions (Chamberlain, Stochl, Redden, & Grant, 2018). *Barratt Impulsiveness Scale, Version 11 (BIS-11)* (three dimensions of impulsivity - attentional, motor, and non-planning) (Patton et al., 1995, Stanford et al., 2016); and the *Cambridge-Chicago Compulsivity Trait Scale (CHI-T)* (compulsive traits) (Chamberlain & Grant, 2018a).

### 2.4 Data analysis

Participants were grouped a priori into current, past or non‐users per the definitions provided above under ‘participants’. Categorical variables were assessed using Pearson’s chi‐square tests. Continuous variables were assessed using Analysis of Variance tests (ANOVA). Effect size was determined using Cramer’s V or Cohen’s D as appropriate. Our primary aim was to show how the groups actually presented, rather than to statistically control for potential covariates, as the former approach is intuitive to clinicians and more likely to be relevant practically both to individuals who use hallucinogens and to healthcare professionals seeing such people. SPSS was used for all statistical analyses (version 24; IBM Corp). Raw p values were reported but findings were only deemed statistically significant if they withstood Bonferroni correction at p < 0.05 two-tailed for the number of measures within a given category of interest (i.e. per table of results).

Missing data were missing completely at random (MCAR) and the analysis was conducted using listwise deletion. By far the most common approach to the missing data is to simply omit those cases with the missing data and analyze the remaining data. This approach is known as the complete case (or available case) analysis or listwise deletion. Listwise deletion is the most frequently used method in handling missing data. Although this may introduce bias in the estimation of the parameters, if the assumption of MCAR is satisfied, a listwise deletion is known to produce unbiased estimates and conservative results. Also, because this was a large sample, where power was not an issue, the assumption of MCAR was satisfied and listwise deletion was thus appropriate.

## 3 Results

Of the 3525 university students (57.7% female) the overall prevalence of past 12-month hallucinogen use was 4.7%, while an additional 6.4% reported lifetime use but not in the past year. Demographic characteristics of the groups are presented in Table 1. It can be seen that those who reported use (ever use/past year use) of hallucinogens were more likely to be Caucasian and had significantly lower educational achievement scores (i.e. lower GPAs).

**Table 1:** Demographics of university students based on use of hallucinogens.

Variable	Students who currently use Hallucinogens (n = 167)	Students who have used Hallucinogens in the past (n = 227)	Students who have never used Hallucinogens (n = 3131)	Statistic Likelihood Ratio	P-Value	Effect Size Cramer’s V
Sex, female, n (%)	84 (51.5)	105 (48.6)	1848 (62.2)	LR = 22.116 df = 6	0.001*	0.058

Year in college, n (%)						
• *Undergraduate*	147 (88.0)	122 (53.7)	2053 (65.6)	LR = 58.791	<0.001*	0.086
• *Graduate*	20 (12.0)	104 (45.8)	1059 (33.8)	df = 4		
• *Non-degree*	0 (0.0)	1 (0.4)	19 (0.6)			
Race/ethnicity, Caucasian	137 (84.6)	178 (82.4)	2216 (74.6)	LR = 15.118 df = 2	0.001*	0.065
Full time student, n (%)	157 (94.0)	192 (84.6)	2898 (92.6)	LR = 16.172 df = 2	<0.001*	0.075

Grade Point Average, GPA						
Less than 3.00	37 (22.2)	33 (14.5)	292 (9.4)	LR = 26.258	<0.001*	0.096
3.00 or higher	130 (77.8)	194 (85.5)	2799 (90.6)	df = 2		

* p < 0.05, Bonferroni corrected.

Hallucinogen use was significantly associated with higher levels of problematic alcohol and illicit substance use (AUDIT and DAST-10). In addition, hallucinogen use was significantly associated with a greater likelihood of using numerous substances, in fact every category of substance for which they were screened (see Table 2).

**Table 2:** Alcohol, tobacco, and illicit drug use in students based on use of hallucinogens.

Variable	Students who currently use Hallucinogens (n = 167)	Students who have used Hallucinogens in the past (n = 227)	Students who have never used Hallucinogens (n = 3131)	Statistic Likelihood Ratio	P-Value	Effect Size Cramer’s V
Age at first use of cigarettes or nicotine						
• Never used	21 (12.6)	34 (15.0)	2064 (65.9)	LR = 432.337	<0.001*	0.251
• Less than 14 years	24 (14.4)	42 (18.5)	134 (4.3)	df = 6		
• 15–17 years	67 (40.1)	85 (37.4)	395 (12.6)			
• 18 years or older	55 (32.9)	66 (29.1)	537 (17.2)			

Frequency of e-cigarette use						
• Never	38 (26.0)	84 (43.5)	653 (61.4)	LR = 100.250	<0.001*	0.198
• Not within past year	31 (21.2)	58 (30.1)	210 (19.7)	df = 8		
• Rarely	53 (36.3)	34 (17.6)	145 (13.6)			
• Occasionally	14 (9.6)	13 (6.7)	34 (3.2)			
• Daily	10 (6.8)	4 (2.1)	22 (2.1)			

Frequency of alcohol consumption						
• Never	6 (3.6)	11 (4.8)	646 (20.6)	LR = 180.58	<0.001*	0.158
• Monthly or less	9 (5.4)	29 (12.8)	632 (20.2)	df = 8		
• 2–4 times a month	59 (35.3)	73 (32.2)	1003 (32.1)			
• 2–3 times a week	61 (36.5)	74 (32.6)	668 (21.3)			
• 4 + times a week	32 (19.2)	40 (17.6)	180 (5.8)			
AUDIT score ≥ 8 (%)	100 (59.9)	106 (46.7)	662 (21.2)	LR = 165.551 df = 2	<0.001*	0.233
DAST-10 score ≥ 3 (%)	90 (53.9)	75 (33.0)	125 (4.0)	LR = 435.113 df = 2	<0.001*	0.452

Non-prescription amphetamines						
• Never	146 (87.4)	187 (83.5)	3108 (99.3)	LR = 209.593	<0.001*	0.266
• In past, not within past 12 months	4 (2.4)	32 (14.3)	9 (0.3)	df = 8		
• Rarely	10 (6.0)	4 (1.8)	8 (0.3)			
• Occasionally	4 (2.4)	0 (0.0)	3 (0.1)			
• Daily	3 (1.8)	1 (0.4)	1 (0.0)			

Cocaine						
• Never	86 (51.8)	104 (46.8)	3045 (97.6)	LR = 740.374	<0.001*	0.466
• In past, not within past 12 months	23 (13.9)	97 (43.7)	42 (1.3)	df = 6		
• Rarely	45 (27.1)	20 (9.0)	24 (0.8)			
• Occasionally	12 (7.2)	1 (0.5)	8 (0.3)			
• Daily	0 (0.0)	0 (0.0)	0 (0.0)			

Opiates						
• Never	151 (90.4)	198 (88.4)	3118 (99.7)	LR = 164.873	<0.001*	0.238
• In past, not within past 12 months	6 (3.6)	25 (11.2)	6 (0.2)	df = 8		
• Rarely	5 (3.0)	0 (0.0)	1 (0.0)			
• Occasionally	2 (1.2)	0 (0.0)	1 (0.0)			
• Daily	3 (1.8)	1 (0.4)	1 (0.0)			

Inhalants						
• Never	145 (56.8)	205 (91.9)	3111 (99.6)	LR = 142.933	<0.001*	0.207
• In past, not within past 12 months	15 (9.0)	17 (7.6)	7 (0.2)	df = 6		
• Rarely	7 (4.2)	1 (0.4)	2 (0.1)			
• Occasionally	0 (0.0)	0 (0.0)	3 (0.1)			
• Daily	0 (0.0)	0 (0.0)	0 (0.0)			

Sedatives						
• Never	113 (67.7)	161 (71.6)	3078 (98.4)	LR = 381.561	<0.001*	0.325
• In past, not within past 12 months	21 (12.6)	47 (20.9)	26 (0.8)	df = 8		
• Rarely	17 (10.2)	12 (5.3)	13 (0.4)			
• Occasionally	13 (7.8)	2 (0.9)	9 (0.3)			
• Daily	3 (1.8)	3 (1.3)	1 (0.0)			

Marijuana						
• Never	3 (1.8)	3 (1.3)	2151 (68.7)	LR = 965.118	<0.001*	0.410
• In past, not within past 12 months	7 (4.2)	76 (33.6)	299 (9.6)	df = 8		
• Rarely	24 (14.4)	59 (26.1)	388 (12.4)			
• Occasionally	76 (45.5)	60 (26.5)	249 (8.0)			
• Daily	57 (34.1)	28 (12.4)	43 (1.7)			

Prescription pain medication						
• Never	103 (62.0)	120 (53.6)	3030 (96.9)	LR = 507.424	<0.001*	0.366
• In past, not within past 12 months	30 (18.1)	87 (38.8)	69 (2.2)	df = 8		
• Rarely	26 (15.7)	13 (5.8)	21 (0.7)			
• Occasionally	4 (2.4)	3 (1.3)	5 (0.2)			
• Daily	3 (1.8)	1 (0.4)	1 (0.0)			

Data refer to N (percentage).

* p < 0.05, Bonferroni corrected.

Table 3 presents the sexual behavior of participants. Hallucinogen use was significantly associated with being sexually active at a younger age and engaging in sex more frequently, and without barrier contraception.

**Table 3:** Sexual behavior in university students based on use of hallucinogens.

Variable	Students who currently use Hallucinogens (n = 167)	Students who have used Hallucinogens in the past (n = 227)	Students who have never used Hallucinogens (n = 3131)	Statistic Likelihood Ratio	P-Value	Effect Size Cramer’s V
Has been sexually active						
• Yes	151 (90.4)	214 (95.1)	2185 (70.5)	LR = 117.072	<0.001*	0.162
• No	16 (9.6)	11 (4.9)	916 (29.5)	df = 2		

Age at first sexual activity with another						
• <11 years	2 (1.3)	2 (0.9)	17 (0.8)	LR = 85.636	<0.001*	0.128
• 12–14 years	17 (11.3)	30 (14.0)	110 (5.0)	df = 8		
• 15–17 years	77 (51.0)	116 (54.2)	867 (39.8)			
• 18–20 years	49 (32.5)	59 (27.6)	880 (40.4)			
• 21 years or older	6 (4.0)	7 (3.3)	306 (14.0)			

Frequency of physical barrier use						
• <50% of the time	67 (44.4)	102 (47.7)	814 (37.4)	LR = 34.062	<0.001*	0.080
• 50–75% of the time	20 (13.2)	25 (11.7)	187 (8.6)	df = 6		
• 76–95% of the time	26 (17.2)	41 (19.2)	352 (16.2)			
• 96–100% of the time	38 (25.2)	46 (21.5)	824 (37.9)			

Data refer to N (percentage).

* p < 0.05 Bonferroni corrected.

Results from specific mental health screens are presented in Table 4. Hallucinogen use was significantly associated with higher rates of depression, PTSD, ADHD, and anxiety. In addition, those who used hallucinogens were more likely to report poorer self-esteem. Hallucinogen use was not significantly associated with gambling disorder, binge-eating disorder, or higher caffeine use.

**Table 4:** Impulsive behaviors and psychiatric history of university students based on use of hallucinogens.

Variable	Students who currently use Hallucinogens (n = 167)	Students who have used Hallucinogens in the past (n = 227)	Students who have never used Hallucinogens (n = 3131)	Statistic Likelihood Ratio	P-Value	Effect Size Cramer’s V
Amount of caffeinated soft drinks consumed over the past week n (%)						
• Never	83 (50.0)	97 (43.7)	1485 (48.3)	LR = 15.822	0.105	0.050
• 1–2 drinks	47 (28.3)	84 (37.8)	988 (32.1)	df = 10		
• 3–6 drinks	22 (13.3)	19 (8.6)	401 (13.0)			
• 7–12 drinks	7 (4.2)	11 (5.0)	138 (4.5)			
• 13–23 drinks	4 (2.4)	7 (3.2)	45 (1.5)			
• 24 or more drinks	3 (1.8)	4 (1.8)	20 (0.6)			
Gambling disorder? • Positive screen	4 (16.7)	0 (0.0)	10 (4.0)	LR = 7.276 df = 2	0.026	0.175
Binge eating disorder? • Positive screen	2 (1.2)	9 (4.1)	72 (2.4)	LR = 3.390 df = 2	0.184	0.032
Has been treated for drug/alcohol use problems • Yes	12 (7.3)	18 (8.1)	32 (1.0)	LR = 54.789 df = 2	<0.001*	0.180
Has been treated for psychological/emotional problems • Yes	75 (45.5)	101 (45.7)	851 (27.8)	LR = 48.405 df = 2	<0.001*	0.123
Currently taking prescribed mental health medication(s) • Yes	35 (21.2)	49 (22.2)	392 (12.8)	LR = 20.586 df = 2	<0.001*	0.082
PHQ-9 Total • Score of 10 or more	42 (26.1)	44 (20.0)	405 (13.4)	LR = 22.654 df = 2	<0.001*	0.087
PTSD • Positive screen	42 (26.1)	44 (20.0)	405 (13.4)	LR = 22.654 df = 2	<0.001*	0.087

Anxiety total Grouped						
• No Anxiety (score 0–4)	70 (43.5)	107 (49.5)	1772 (59.6)	LR = 25.428	<0.001*	0.062
• Mild (score 5–9)	55 (34.2)	68 (31.5)	686 (23.1)	df = 6		
• Moderate (score 10–14)	24 (14.9)	23 (10.6)	325 (10.9)			
• Severe (score 15–21)	12 (7.5)	18 (8.3)	192 (6.5)			
ADHD • Positive screen	48 (29.4)	60 (27.6)	482 (16.1)	LR = 31.573 df = 2	<0.001*	0.103
Rosenberg Self-esteem scale • Less than 15	39 (24.4)	28 (13.1)	425 (14.4)	LR = 10.892 df = 2	0.004*	0.061

Data refer to N (percentage).

* p < 0.05 Bonferroni corrected.

In terms of psychological traits, those who used hallucinogens reported significantly greater scores of impulsivity on all subscales of the BIS-11, but did not report greater levels of compulsive traits on the CHI-T (see Table 5).

**Table 5:** Impulsivity and compulsivity of university students based on use of hallucinogens.

Variable	Students who currently use Hallucinogens (n = 167)	Students who have used Hallucinogens in the past (n = 227)	Students who have never used Hallucinogens (n = 3131)	Statistic ANOVA	P-Value	Effect Size Cohen’s d
Cambridge-Chicago Compulsivity Trait Scale Mean (sd)	11.18 (14.13)	9.04 (13.28)	9.24 (13.5)	F (2,3413) = 1.622	0.198	0.052
Barratt Impulsiveness Scale (BIS-11) Total Score Mean (sd)	65.42 (9.5)^a^	63.09 (10.1)^b^	58.86 (10.08)^ab^	F (2,3186) = 46.491	<0.001*	0.522
Attentional impulsiveness Mean (sd)	17.81 (3.86)^a^	17.55 (4.04)^b^	16.03 (3.95)^ab^	F (2,3279) = 28.470	<0.001*	0.414
Non-planning impulsiveness Mean (sd)	35.31 (4.66)^a^	24.13 (4.45)^b^	22.73 (4.74)^ab^	F (2,3273) = 29.769	<0.001*	0.405
Motor impulsivenessMean (sd)	22.37 (4.19)^a^	21.53 (3.93)^b^	20.13 (3.91)^ab^	F (2,3286) = 35.623	<0.001*	0.449

Data refer to mean and (standard deviation).

ab Post Hoc Bonferroni Test for Significance: The mean difference is significant at the 0.05 level.

* p < 0.05 Bonferroni corrected.

## 4 Discussion

This study examined the prevalence of hallucinogen use in a large sample of university students; and ways in which hallucinogen use was related to concomitant use of other drugs as well as mental health and academic achievement. We found that 4.7% of the sample reported past 12-month hallucinogen use (with an additional 6.4% having ever used them). Overall, the lifetime rates found in our study (almost 11.1%) are similar to (although somewhat higher than) those reported in the NESARC study, where 9.32% had used hallucinogens in their lifetimes (Shalit et al., 2019). Based on this study, hallucinogen use appears to be particularly high in young adults, and these findings are concerning regarding the long term effects of this use during young adulthood. Although research conducted in adolescents aged 12–17 years (N = 55,286) suggests that the majority of young people who use hallucinogens do not develop a hallucinogen use disorder, data did suggest that approximately 30% of past-year hallucinogen users reported symptoms of a hallucinogen use disorder and that 17% of hallucinogen users met criteria for a past-year hallucinogen use disorder (Wu, Ringwalt, Weiss, & Blazer, 2009). Which of these young adults will have future problems with a hallucinogen use disorder is not, however, known to be predictable on the individual level.

Clearly, young adults who use hallucinogens also use and have problems with a range of addictive substances and unhealthy behaviors. One possible explanation is that a common cognitive/personality feature underlies all of these problems associated with hallucinogen use (for example, elevated impulsivity as seen on the BIS-11). Alternatively, the use of various drugs may result in neurobiological changes that predispose a young person to becoming impulsive. There are limited data regarding adverse neurobiological effects of hallucinogens based on amount of drug used, frequency of use, and age of initiation of use. If either of the above is true, at least for some young adults, then addressing the underlying impulsivity would be potentially more beneficial than directly addressing each problematic behavior.

Another, non-mutually exclusive explanation for the association of hallucinogen use with using a variety of drugs and with impulsive behaviors and tendencies could be that various mental health problems (e.g., depression, PTSD, etc.) give rise to young adults attempting to self-medicate their emotional states with a variety of drugs, including hallucinogens. This theory has led many to examine whether hallucinogens may offer a rapid treatment for depression and other mental health problems (Bogenschutz et al., 2015, Carhart-Harris et al., 2017, de Gregorio et al., 2018).

Interestingly, we found that participants who used hallucinogens reported worse depressive and anxiety symptoms, than those who had never used hallucinogens, with no differences between current or past users. Thus, these data fail to produce compelling evidence that hallucinogens may be working as antidepressants or anxiolytics in this ecological setting.

This study into the use of hallucinogens has the advantage of being relatively large. Nonetheless, there are several limitations that should be considered. The study was cross-sectional and hence the direction of causality of any effects cannot be established – this would require longitudinal research on the topic; however, we hope that such cross-sectional data will support such follow-up. There are also limitations inherent in the study being conducted using an online interface via the Internet – diagnostic assessment may be less accurate via such an online survey compared to in-person assessment by a clinician; there may be responder biases; and there may be under-reporting (though this possibility is reduced by individuals’ responses not being lacked to personally identifiable information). Additionally, self-report questions pertaining to substance use and other potentially socially embarrassing behaviors e.g. multiple sexual partners have their own limitations: for example, individuals may not disclose the full extent of their actions or may not report it accurately due to bias.

In summary, we found in a large sample of university students that hallucinogens use was common, and associated with drug use and a number of mental health problems, plus higher impulsivity.

## Contributors

Dr. Grant designed the study, wrote the protocol, and conducted literature searches.

Dr. Lust conducted the statistical analysis.

Dr. Chamberlain co-wrote the first draft of the manuscript.

All authors contributed to the final manuscript submission.

## Role of funding sources

This research was funded by internal funds. Dr Chamberlain’s role in this study was funded by a Wellcome Trust Clinical Fellowship (Reference 110049/Z/15/Z).

## Declaration of Competing Interest

Dr Grant has received research grants from National Center for Responsible Gaming, and Promentis and Otsuka Pharmaceuticals Dr Grant receives yearly compensation from Springer Publishing for acting as Editor-in-Chief of the Journal of Gambling Studies and has received royalties from Oxford University Press, American Psychiatric Publishing, Inc., Norton Press, and McGraw Hill. Dr Chamberlain consults for Cambridge Cognition, Shire, Promentis, and Ieso. Dr Chamberlain receives a stipend for his role as Associate Editor at Neuroscience and Biobehavioral Reviews; and at Comprehensive Psychiatry. Dr. Lust has no conflicts to report.
